# Acalculous Cholecystitis as a Complication of Primary Epstein-Barr Virus Infection: A Case-Based Scoping Review of the Literature

**DOI:** 10.3390/v16030463

**Published:** 2024-03-18

**Authors:** Aristotelis Tsiakalos, Georgios Schinas, Aggelos Karatzaferis, Emmanouil Angelos Rigopoulos, Christos Pappas, Eleni Polyzou, Effrosyni Dimopoulou, George Dimopoulos, Karolina Akinosoglou

**Affiliations:** 1 Leto General, Maternity-Gynecological Clinic, 11524 Athens, Greece; atsiakalos@gmail.com; 2 School of Medicine, University of Patras, 26504 Rio, Greece; georg.schinas@gmail.com (G.S.); agrigopoulos@gmail.com (E.A.R.); polyzou.el@gmail.com (E.P.); 3 Alpha Bank SA, 10564 Athens, Greece; angelos.karatzaferis@alpha.gr; 4 Euroclinic Athens, 11521 Athens, Greece; chrispap80@yahoo.gr; 5 Hellenic Institute for the Study of Sepsis, 11528 Athens, Greece; efidimop98@gmail.com; 6 3rd Department of Critical Care, Evgenidio Hospital, Medical School, National and Kapodistrian University of Athens, 11528 Athens, Greece; gdimop@med.uoa.gr; 7 Department of Internal Medicine and Infectious Diseases, University General Hospital of Patras, 26504 Rio, Greece

**Keywords:** Epstein-Barr virus, acalculous cholecystitis, ascites, Gilbert syndrome, infectious mononucleosis, hepatobiliary complications

## Abstract

Primary Epstein-Barr virus (EBV) infection manifests with diverse clinical symptoms, occasionally resulting in severe complications. This scoping review investigates the rare occurrence of acute acalculous cholecystitis (AAC) in the context of primary EBV infection, with a focus on understanding its prevalence, clinical features, and underlying mechanisms. The study also explores EBV infection association with Gilbert syndrome, a condition that potentially exacerbates the clinical picture. Additionally, a case report of an 18-year-old female presenting with AAC and ascites secondary to EBV infection enhances the review. A comprehensive literature review was conducted, analyzing reported cases of AAC secondary to EBV infection. This involved examining patient demographics, clinical presentations, laboratory findings, and outcomes. The search yielded 44 cases, predominantly affecting young females. Common clinical features included fever, cervical lymphadenopathy, tonsillitis/pharyngitis, and splenomegaly. Laboratory findings highlighted significant hepatic involvement. The review also noted a potential link between AAC in EBV infection and Gilbert syndrome, particularly in cases with abnormal bilirubin levels. AAC is a rare but significant complication of primary EBV infection, primarily observed in young females, and may be associated with Gilbert syndrome. This comprehensive review underscores the need for heightened clinical awareness and timely diagnosis to manage this complication effectively.

## 1. Introduction

The clinical spectrum of primary Epstein-Barr virus (EBV) infection is diverse. In pediatric populations, it often manifests as asymptomatic or presents with nonspecific symptoms. In contrast, adolescents and young adults frequently develop the clinical syndrome known as infectious mononucleosis (ΙΜ) [1,2]. Typically, IM starts with a sense of discomfort, headache, and a mild fever before progressing to more specific indications such as tonsillitis and/or pharyngitis, symmetrical swelling and sensitivity in the cervical lymph nodes, and moderate to high fever. Pharyngitis commonly includes tonsil discharge that may appear white, gray-green, or necrotic. Pronounced fatigue might be a significant symptom, while other less frequent observations encompass small hemorrhages in the palate, swelling around the eyes, or skin rashes resembling small spots or measles. Nausea, vomiting, and loss of appetite are common among patients, and are potentially linked to the mild hepatitis observed in roughly 90% of those infected. Enlargement of the spleen occurs in up to 50% of patients; however, jaundice and liver enlargement are infrequent. Less commonly, patients may experience hematological complications (e.g., hemolytic anemia, aplastic anemia, or thrombocytopenia) and neurological manifestations (e.g., meningoencephalitis, Guillain-Barre syndrome, or peripheral neuritis). Additional severe complications such as splenic rupture, upper airway obstruction, and hemophagocytic lymphohistiocytosis have also been reported.

Acute acalculous cholecystitis (AAC) is a complication of primary Epstein-Barr virus infection, mostly observed in young girls and women. Associated risk factors for acalculous cholecystitis include critical illness, trauma, burns, surgery, sepsis, prolonged fasting, total parenteral nutrition, immunodeficiency, chronic illness, and vasculitis, and its pathogenesis likely results from bile stasis and/or ischemia [3]. In the context of EBV, Fretzayas et al. conducted radioisotopic cholangiography in two female patients with acalculous cholecystitis during the course of primary EBV infection, and their findings demonstrated impaired function of gallbladder emptying with low ejection fraction, while the gallbladder filling process was normal. These results were suggestive of primary dyskinesia [4]. Another possible pathophysiological mechanism involves direct invasion of the gallbladder by the virus, as has been reported in a case of cholecystitis caused by hepatitis A virus through immunostaining [5].

Nevertheless, the majority of individuals with primary EBV infection recover fully and acquire long-lasting immunity. Acute symptoms typically subside within one to two weeks, but fatigue often lingers for several weeks to months. Patients suspected of having infectious mononucleosis, based on their medical history and physical examination, should undergo a white blood cell count with differential testing and a heterophile test, like the Monospot test, or EBV-specific antibody testing. Additionally, patients should be evaluated for streptococcal infection through culture or antigen testing. In a patient displaying symptoms consistent with the syndrome but testing negative for heterophile antibodies, it may be advisable to repeat the Monospot test, as it could yield a negative result during the initial week of illness. Alternatively, or in conjunction, EBV-specific antibodies (including IgM and IgG antibodies targeting viral capsid antigen [VCA], and IgG antibodies targeting nuclear antigen and early antigen) can be analyzed. EBV-specific antibodies are especially beneficial if the patient consistently tests negative on the Monospot test. The presence of EBV nuclear antigen (EBNA) IgG antibodies, or the absence of both IgG and IgM antibodies to VCA, rules out an acute primary EBV infection and warrants consideration of other causes for a mononucleosis-like illness, such as cytomegalovirus (CMV), primary HIV infection, and toxoplasmosis. Advanced molecular methods, such as polymerase chain reaction (PCR), may be utilized for resolving unclear serology results and for latter phases of the disease, such as 2 weeks after illness onset [6]. PCR testing of serum in pediatric and young adult patients with infectious mononucleosis demonstrates high specificity (98%) and good sensitivity (77%) for the detection of EBV DNA [7].

In light of these considerations, the primary aim of this study is to elucidate the prevalence, clinical characteristics, and management strategies of AAC in the context of primary EBV infection. Additionally, this research seeks to explore the association between this rare complication and Gilbert syndrome, aiming to provide a clearer understanding of any potential exacerbating effects Gilbert syndrome may have on the clinical picture of AAC. Ultimately, by combining a detailed case report with an extensive literature review, this study seeks to contribute to the growing body of research on the subject and highlight the importance of considering AAC in patients presenting with primary EBV infection.

## 2. Case Report

We hereby describe a case involving an 18-year-old female who presented with AAC and ascites secondary to EBV infection and provide a scoping review of the available literature on the subject. This study was conducted in full compliance with the ethical principles of the Declaration of Helsinki and Good Clinical Practice guidelines. The study took place at Sotiria Hospital’s Third Department of Internal Medicine, and informed consent was obtained from the patient involved in the case report.

An 18-year-old female with no prior medical history presented to the emergency department with a fever peaking at 39.5 °C, accompanied by a 6-day history of sore throat, nausea, vomiting, and progressively worsening diffuse abdominal pain. Vital signs were within normal limits: blood pressure 120/80 mmHg, heart rate 90/min, and O_2_ saturation 98% on ambient room air (FiO_2_ 21%). Physical examination revealed cervical lymphadenopathy, scleral icterus, and unilateral tonsillar enlargement. Abdominal examination demonstrated right upper quadrant tenderness with a positive Murphy’s sign. Laboratory findings included a complete blood count with a total leukocyte count of 6.1 × 10^3^/μL (49.1% granulocytes, 44.2% lymphocytes, 6.7% monocytes), a hemoglobin level of 15.3 g/dL, and a platelet count of 140 × 10^3^/μL. CRP level was elevated at 7.92 mg/dL (normal upper limit 0.30 mg/dL). Coagulation tests showed an INR of 1.38 and APTT of 33.3 s (normal values 28–40 s). Liver function tests were significantly abnormal: total bilirubin 2.8 mg/dL, direct bilirubin 2.4 mg/dL, AST 468 U/L (normal values 15–37 U/L), ALT 423 U/L (normal values 12–78 U/L), alkaline phosphatase 667 U/L (normal values 50–136 U/L), GGt 423 U/L (normal values 5–85 U/L), and LDH 730 U/L (normal values 81–234 U/L). Atypical lymphocytes were observed on peripheral blood smear, and heterophile antibody testing was negative.

Baseline ultrasonographic examination of the abdomen revealed mild splenomegaly. Imaging of the gallbladder was inconclusive due to gallbladder contraction. Persistent tenderness in the right upper quadrant was noted, along with subsequent elevations in total bilirubin and INR levels, peaking at 5 mg/dL and 1.63, respectively (Table 1).

A follow-up abdominal ultrasound revealed gallbladder wall thickening, pericholecystic edema, and the absence of gallstones. Additionally, significant ascitic fluid was identified in the pouch of Douglas, the uterorectal space, and the right iliac fossa. Empirical antimicrobial therapy with cefoxitin and doxycycline was initiated. Intrapelvic ultrasound and vaginal discharge cultures were performed, revealing no signs of pelvic inflammatory disease and thereby excluding Fitz-Hugh Curtis syndrome as a differential diagnosis. The presence of atypical lymphocytes on peripheral blood smear persisted. EBV infection was confirmed through the detection of IgM antibodies against the viral capsid antigen. Antimicrobial therapy was subsequently discontinued. By the twelfth day post-admission, the patient became afebrile, abdominal tenderness resolved, and a repeat ultrasound showed normalization of the gallbladder. In order to verify the diagnosis of Gilbert syndrome, we performed DNA sequencing of the UGT1A gene. PCR was performed for amplification of exons, and the promoter region and enhancer regions of the UGT1A1 and the amplified DNA fragments were directly sequenced. Genetic testing revealed that the patient was a carrier of the UGT1A1*28 autosomal recessive mutation in the promoter region [A(TA)6TAA] of the uridine diphosphate glucuronyl transferase (UGT)1A1 gene. At a three-month follow-up, the patient remained in excellent clinical condition.

## 3. Scoping Review of the Literature

### 3.1. Materials and Methods

Acute acalculous cholecystitis (AAC) during primary EBV infection is an exceedingly rare complication. To better understand the prevalence and clinical characteristics of this condition, a comprehensive literature review was conducted using the PubMed database. The specific queries employed were “acute acalculous cholecystitis” OR “Cholecystitis” AND “Epstein-Barr virus” OR “EBV”, targeting studies that explicitly discussed AAC in the setting of primary EBV infection. Our inclusion criteria were as follows: (1) patients of any age diagnosed with AAC in the context of primary EBV infection, where the diagnosis was supported by serological evidence indicative of recent infection; (2) reports that included case descriptions detailing clinical presentation, diagnosis, and management of AAC during primary EBV infection; (3) articles identified as case reports, case series, and review articles focusing on clinical cases; and (4) articles published in English. We excluded (1) studies not involving AAC associated with primary EBV infection; (2) reports without full case descriptions (e.g., abstracts or articles without full text availability); and (3) literature reviews. 

**Figure 1:**
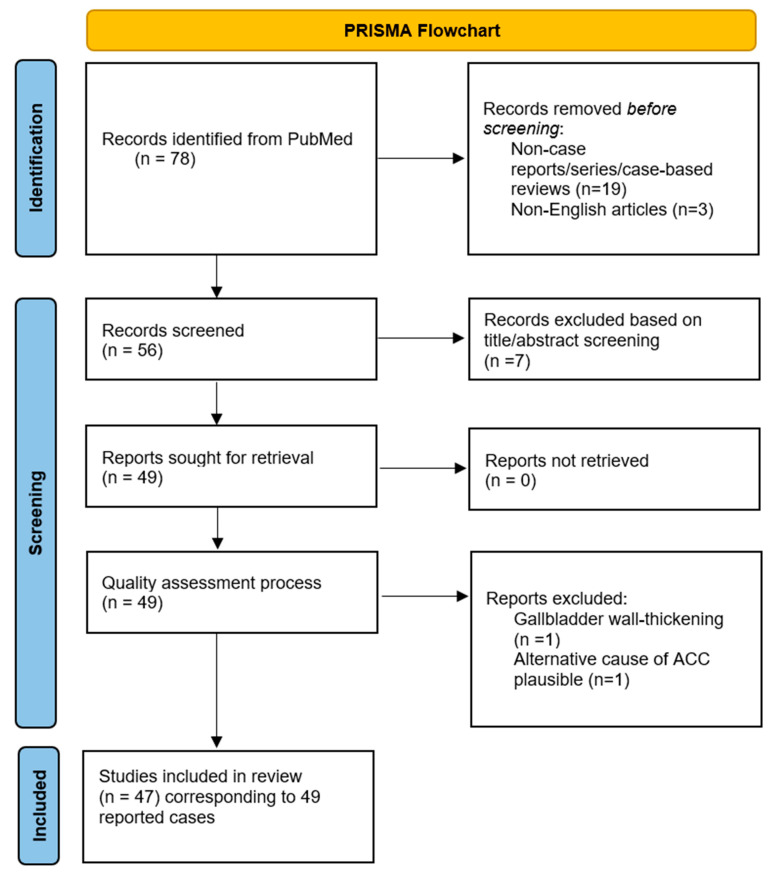
PRISMA diagram.

Our strategy yielded 50 published clinical cases, with the most recent one reported in December 2023. The different phases of this review are diagrammatically represented in Figure 1 as a PRISMA flowchart. Extracted data included (1) patient demographics (e.g., age and sex) and study characteristics (e.g., type of study, country of origin); (2) clinical presentation, including cardinal symptoms of EBV and laboratory findings; (3) management strategies, including medical and surgical interventions; and (4) reporting of Gilbert syndrome and the presence of ascites. In Table 2, we provide a summary of our findings. Descriptive statistics were subsequently employed to present them in a narrative manner.

To evaluate the methodological quality of the selected studies and verify causality between EBV and AAC in the reported cases, we applied the appropriate parts of the quality assessment tool suggested by Murad et al. for case reports/series [8]. This tool assesses several key domains—selection of patient/clinical case, ascertainment of exposure and outcomes, exclusion of alternative causes, follow-up, and reporting details—with specific leading questions guiding evaluation. A detailed description of the methodology employed can be found in Table S1 (Supplementary Materials). Overall, two case reports were excluded for the following reasons: one was related to gallbladder wall-thickening, which did not directly pertain to the primary focus of our study; and another presented an alternative plausible cause for ACC, namely scrub typhus. Every other study included in our review met all of the standards described (Table S2, Supplementary Materials).

**Table 2:** Summary of key characteristics and laboratory findings in published cases of acute acalculous cholecystitis during primary EBV infection.

Authors	Study Design	Country	Age/Sex	Fever	Cervical Lymphadenopathy	Tonsilitis/ Pharyngitis	Spleno-Megaly	AST/ALT/ALP/GGT	Total Bilirubin (Direct)	Ascites	Gilbert Syndrome	Antibiotic Treatment	Surgery
Fretzayas A. et al. [4]	Case-series	Greece	11/Female	+	+	+	+	291/198/536/52	1.80 (0.4)	N/R	+	−	−
Fretzayas A. et al. [4]	Case-series	Greece	12/Female	+	−	−	+	134/125//162	Normal range	N/R	N/R	−	−
Beltrame V. et al. [9]	Case report	Italy	29/Male	+	+	N/R	N/R	121/166/161/145	1.36 (0.73)	N/R	N/R	+	−
Attilakos A. et al. [10]	Case report	Greece	5/Male	+	+	+	+	207/257/919/333	1.8 (0.9)	N/R	+	−	−
Arya S.O. et al. [11]	Case report	USA	16/Female	+	+	+	−	126/39/409/173	2.5 (1.4)	N/R	N/R	+	−
Suga K. et al. [12]	Case report	Japan	6/Female	+	+	+	−	284/139/506/36	0.4 (0.1)	N/R	N/R	−	−
Gagneux-Brunon A. et al. [13]	Case report	France	18/Female	+	−	−	−	321/214/165/64	1.17 (N/R)	N/R	N/R	+	−
Gagneux-Brunon A. et al. [13]	Case report	France	20/Female	+	+	+	−	453/494/133/286	2.23 (N/R)	N/R	N/R	+	−
Iaria C. et al. [14]	Case report	Italy	18/Female	+	+	+	−	220/328/312/142	7.0 (4.26)	N/R	N/R	+	−
Prassouli A. et al. [15]	Case report	Greece	13/Female	+	N/R	+	−	394/674/721/352	4 (3.5)	N/R	N/R	+	−
Cholongitas E. et al. [16]	Case report	Greece	19/Female	+	+	+	−	426/584/710/156	6.5 (5.17)	N/R	N/R	−	−
Lagona E. et al. [17]	Case report	Greece	4/Female	+	+	+	+	188/304/236/241	4.6 (3.6)	N/R	N/R	−	−
Ono S. et al [18]	Case report	Japan	33/Female	+	+	+	−	225/263/1591/174	1.21 (N/R)	N/R	N/R	+	−
Majdalani M. et al. [19]	Case report	Lebanon	16/Female	+	−	−	+	163/136/625/218	2.5 (2.1)	N/R	N/R	+	−
Koufakis T. et al. [20]	Case report	Greece	21/Male	+	−	−	−	172/232/179/350	6.31 (4.96)	N/R	N/R	−	−
Rodà D. et al. [21]	Case report	Spain	2/Male	+	+	+	+	157/501/800/433	N/R	+	N/R	+	−
Branco L. et al. [22]	Case report	Portugal	16/Female	+	+	+	−	340/689/224/271	0.7 (0.3)	N/R	N/R	+	−
Pawłowska-Kamieniak A. et al. [23]	Case report	Poland	17/Female	+	−	+	−	230/268/311/135	2.2 (N/R)	N/R	N/R	+	−
Alkhoury F. et al. [24]	Case report	USA	15/Female	+	−	−	−	191/221/221/(N/R)	1.8 (1.5)	+	N/R	−	−
Agergaard J. et al. [25]	Case report	Denmark	34/Female	+	−	+	−	(N/R)/61/737/42	N/R	N/R	N/R	+	−
Koch A. D. et al. [26]	Case report	Netherlands	53/Female	+	N/R	N/R	N/R	422/339/1081/(N/R)	7.02 (N/R)	N/R	N/R	−	−
Yang H. N. et al. [27]	Case report	Korea	20/Female	+	+	+	+	171/299/727/202	0.7 (N/R)	N/R	N/R	+	−
Rezkallah KN. et al. [28]	Case report	USA	25/Female	+	−	−	−	94/116/154/(N/R)	N/R (0.5)	N/R	N/R	−	+
Yoshie K. et al. [29]	Case report	Japan	15/Female	+	+	+	+	147/215/569/(N/R)	N/R	N/R	N/R	−	−
Pelliccia P. et al. [30]	Case report	Italy	14/Female	+	+	+	+	137/108/353/ (N/R)	N/R	+	N/R	−	−
Hagel S. et al [31]	Case report	Germany	21/Female	+	N/R	N/R	+	N/R	2.51 (N/R)	N/R	N/R	+	+
Nagdev A. et al. [32]	Case report	USA	18/Female	+	N/R	−	N/R	118/(N/R)/146/(N/R)	1.2 (0.6)	N/R	N/R	+	−
Carrascosa MF. et al. [33]	Case report	Spain	22/Female	+	+	N/R	+	329/464/239/(N/R)	2.49 (2.39)	+	N/R	−	−
Strehle E. et al. [34]	Case report	UK	14/Female	+	N/R	N/R	N/R	207/(N/R)/178/111	N/R (2.34)	N/R	N/R	+	−
Sheybani F. et al. [35]	Case report	Iran	23/Female	+	+	+	+	169/641/909/(N/R)	2.3 (1.1)	+	N/R	−	−
Yesilbag Z. et al. [36]	Case report	Turkey	30/Female	+	N/R	−	+	233/220/376/471	15.4 (14.5)	N/R	N/R	+	−
Cameron A. et al. [37]	Case report	Canada	18/Female	+	+	+	N/R	461/671/258/(N/R)	2.05 (N/R)	N/R	N/R	−	−
Höhn P. et al. [38]	Case report	Germany	24/Male	+	+	N/R	−	116/185/437/258	N/R	N/R	N/R	−	−
Boninsegna S. et al. [39]	Case report	Italy	24/Female	+	N/R	N/R	+	919/914/(N/R)/(N/R)	4.4 (3)	N/R	N/R	+	−
Young C. et al. [40]	Case	USA	14/Female	+	−	−	−	22/13/(N/R)/(N/R)	0.2 (N/R)	N/R	N/R	+	−
Ntelis K. et al. [41].	report	Greece	15/Female	+	+	+	+	106/217/421/177	0.84 (0.32)	N/R	N/R	+	−
Suda T. et al. [42]	Case report	Japan	34/Female	+	+	+	+	368/450/620/275	2.1 (N/R)	N/R	N/R	−	−
Langenohl R. et al. [43].	Case report	USA	3/Male	+	+	N/R	+	259/247/(N/R)/(N/R)	4.3 (N/R)	+	N/R	+	−
Leganés Villanueva C. et al. [44].	Case report	Spain	10/Female	+	+	+	+	279/301/642/297	29 (10)	N/R	N/R	−	−
Nakagawa H. et al. [45].	Case report	Japan	20/Female	N/R	+	+	+	201/190/433/132	N/R	N/R	N/R	−	−
Harvey KG. et al. [46].	Case report	USA	17/Female	N/R	N/R	+	+	105/80/174/(N/R)	4.8 (N/R)	N/R	N/R	−	−
Rein J. et al. [47]	Case report	USA	7/Female	+	+	N/R	+	(N/R)/114/(N/R)/(N/R)	1.8 (1.1)	N/R	N/R	+	−
Avcu G. et al. [48]	Case report	Turkey	15/Female	+	−	+	+	178/268/233/203	2.5 (1.96)	N/R	N/R	+	−
Celik F. et al. [49]	Case report	Turkey	48/Female	+	+	N/R	N/R	221/165/516/224	14.43 (12.9)	N/R	N/R	+	−
Teopoulos Lamprianidis K. et al. [50].	Case report	UK	20/Female	+	+	+	+	(N/R)/247/840/(N/R)	6.8 (N/R)	+	N/R	−	−
Barkho F. et al. [51].	Case Report	USA	19/Male	+	+	N/R	+	150/218/121/(N/R)	3.2 (N/R)	N/R	N/R	−	−
Trbojević T. et al. [52]	Case Report	Croatia	5/Female	−	N/R	−	+	1908/3222/385/51	4.89 (4.0)	N/R	(Family history negative)	−	−
Teles H. et al. [53]	Case Report	Portugal	10/Female	+	+	+	N/R	240/31/(N/R)/(N/R)	N/R	N/R	N/R	+	−
Khan U et al. [54].	Case Report	Norway	Late teens/Female	N/R	N/R	+	N/R	(N/R)/416/218/256	0.8 (0.7)	N/R	N/R	+	−
Present case	Case report	Greece	28/Female	+	+	+	+	468/423/667/423	2.8 (2.4)	+	+	+	−

AST: aspartate aminotransferase, ALT: alanine aminotransferase, ALP: alkaline phosphatase, GGT: gamma-glutamyl transferase. [(+): presence; (−): absence; N/R: not reported].

### 3.2. Results

This comprehensive literature review, encompassing 50 reported cases (including the one reported in this article), offers insights into the clinical and laboratory characteristics of acalculous cholecystitis in the context of EBV infection, as well as the outcomes of affected patients. 

In this review, a marked gender discrepancy was evident: a significant majority of the cases involved females (44/50). The age of the individuals in the reviewed cases ranged from 2 to 53 years, demonstrating that acalculous cholecystitis associated with EBV infection can manifest across a broad age spectrum. However, the median age of 17 years suggests a predominance in adolescents and young adults. Interestingly, our patient was heterozygous for the UGT1A1 gene mutation. Gilbert syndrome was explicitly identified in three cases, with one additional report noting a negative family history for the syndrome. This observation suggests a low reported prevalence of Gilbert syndrome among the reported cases, which may influence the clinical interpretation of bilirubin levels and warrants careful consideration in the management of AAC in the context of EBV infection. AAC is a very rare complication, with the majority of recently described cases occurring in Caucasians, primarily from southern Europe (France, Greece and Italy). Notably, reports from Greece accounted for nine cases, while five case reports were sourced from Italy. This observation could be correlated with the increased incidence of Gilbert syndrome (GS) in the Italian and Greek populations (16.9% and 18.6% respectively) [55]. Attilakos et al. [10] reported a possible correlation between the occurrence of AAC and GS in children with infectious mononucleosis due to EBV. This phenomenon could be explained through the decreased hepatic glucuronidase activity observed in GS patients, which contributes to cholestasis during the course of EBV infection [55]. 

In terms of clinical manifestations, our review demonstrated that fever was the predominant symptom, observed in nearly all cases that reported its occurrence (46/47). Cervical lymphadenopathy and tonsillitis/pharyngitis were also common, documented in 30/40 and 28/39 cases that provided information on these symptoms, respectively. Conversely, splenomegaly was identified in approximately 60% of the cases (26/42) that noted its occurrence, suggesting that the absence of this condition should not automatically exclude the possibility of this complication. The current case, involving an 18-year-old female, aligns closely with the trends observed in the literature. The patient presented with fever, cervical lymphadenopathy, tonsillitis/pharyngitis, and splenomegaly, features commonly observed in the reviewed cases. A significant difference in our patient’s case was the simultaneous development of ascites during the course of the illness. There are limited reports describing patients with ascites during EBV mononucleosis, and those that do exist describe specific patient types such as immunosuppressed children with primary EBV infection [56], very young children [21], and children with combined EBV and CMV infection [57]. Ascites was less commonly reported in literature, occurring in only eight other cases. Although these symptoms often align with the general symptomatology of EBV infections, their pattern indicates a predisposition for severe disease manifestations, potentially leading to complications such as acalculous cholecystitis. Our case is unique since it describes both acalculous cholecystitis and ascites during the course of a primary EBV infection in an adult individual, where the ascites was not associated with hypoproteinemia. The presence of these two less frequent features adds complexity to the clinical management required and might suggest a more severe disease course.

Laboratory findings further corroborated the hepatic involvement commonly seen in severe EBV infections. Significant hepatic involvement was noted in the majority of cases, with wide variability in AST, ALT, ALP, and GGT levels, indicative of a mixed pattern of hepatocellular injury and cholestasis. Bilirubin levels also varied among reported cases. In 26/43 cases that provided information on bilirubin laboratory parameters, the total bilirubin level was over 2 mg/dL, indicating significant hyperbilirubinemia in more than half of the reported cases. 

Antibiotics were administered in the majority of cases (27/50), reflecting either the suspected presence of secondary infection or a precautionary measure against potential bacterial superinfection, while surgery was noted to be a rare intervention, with only two cases undergoing surgical treatment, emphasizing the general approach of conservative management in AAC associated with EBV infection.

Overall, our review findings highlight AAC as a significant but rare complication of primary EBV infection, with a global incidence and a female predominance. The clinical presentation is diverse, with the classic triad of cardinal symptoms, e.g., fever, cervical lymphadenopathy, and tonsillitis/pharyngitis being very common. Correlation with Gilbert’s syndrome could not be established. Laboratory findings reveal significant liver involvement, with varying degrees of enzyme elevations and sometimes hyperbilirubinemia. Finally, the management largely leans conservative, with antibiotics being the mainstay of treatment. 

Despite the comprehensive nature of our review in elucidating the prevalence and clinical characteristics of AAC during primary EBV infection, some limitations warrant mention. First, the reliance on published case reports and series inherently limits the generalizability of our findings, as these types of studies may be subject to publication and reporting bias, where only cases with unusual presentations or outcomes are reported. This selective publication and reporting could skew our understanding towards more dramatic manifestations of acute acalculous cholecystitis (AAC) in the context of primary Epstein-Barr virus (EBV) infection, potentially misrepresenting its true clinical spectrum. Lastly, the lack of longitudinal follow-up in many case reports limits insights into long-term outcomes and the potential for recurrent episodes of AAC in patients with primary EBV infection.

## 4. Discussion

### 4.1. Virology and Epidemiology of EBV 

EBV serves as the causative factor for infectious mononucleosis. In most adults, EBV remains a latent infection without symptoms throughout life. However, it is linked with the occurrence of various cancers such as B cell lymphoma, T cell lymphoma, Hodgkin lymphoma, and nasopharyngeal carcinoma. When primate B lymphocytes are infected, it typically results in a latent infection. This state is recognized by the continuous existence of the viral genome, accompanied by the expression of a limited range of latent gene products, contributing to the transformation process and promoting cell proliferation. The majority of initial EBV infections in humans are believed to begin in the oropharynx. Oropharyngeal epithelial cells allow viral replication, unlike B lymphocytes. Viral latency involves three specific processes, including sustained viral presence, restricted viral gene expression, and the potential for reactivation leading to lytic replication. Prolonged EBV infection might occur due to the virus’s strategies to evade host immune responses. EBV-infected latent B cells are considered to be oncogenically transformed since they proliferate endlessly when cultured in vitro. These cells can also generate lymphoproliferative disorders, including lymphoma, in individuals with either congenital or acquired immunodeficiency.

It Is transmitted through close contact between individuals susceptible to the virus and those actively shedding EBV. The absence of the virus in environmental sources implies that humans serve as the primary reservoir. Antibodies to EBV have been detected across all population groups worldwide, with around 95% of adults eventually testing positive for EBV. By the age of four, nearly all children in resource-limited countries acquire EBV, while in lower socioeconomic groups in the United States, the seroprevalence ranges from 25–50% [58]. As a matter of fact, according to data from the U.S. National Health and Nutrition Examination Survey (NHANES) for children aged 6–19 from 2003–2010, children in the lowest income quartile had a substantially higher seroprevalence (81.0%) compared to those in the highest income quartile (53.9%) [59]. EBV infections contracted during childhood often manifest as subclinical; even with high exposure rates, fewer than 10% of children show clinical infection. The occurrence of symptomatic infection becomes more prevalent during adolescent and adult years, with the typically noted peak incidence of infection remaining within the 15 to 24-year age range [60]. Recent data from the United Kingdom indicate potential occurrences of infectious mononucleosis in later life, with increased severity necessitating hospitalization [61]. In adults, infectious mononucleosis is relatively rare, contributing to less than 2% of pharyngitis cases. The majority of adults are immune to this infection due to previous exposure. The distinctions observed between symptomatic infections in infants and young adults remain unclear. Possible reasons may include the viral inoculum size at the time of infection, or the strength of immune responses directed by EBV-infected B cells. The reasons behind why some children and adolescents develop infectious mononucleosis while others do not are unknown, with hypotheses suggesting that variations in single-nucleotide polymorphisms within toll-like receptors might contribute to the varying courses of acute, primary EBV infections [62]. Moreover, genetic factors might influence who develops the clinical disease. In a particular case series, GATA2 deficiency was linked to severe primary EBV infections requiring hospitalization or hemophagocytic lymphohistiocytosis with lymphoma, indicating that this genetic deficiency might influence disease presentation in certain instances [63]. 

### 4.2. Clinical Course and Complications 

Infectious mononucleosis stands out as the primary acute clinical presentation of EBV [64,65]. Typical symptoms include fever, pharyngitis, lymphadenopathy, and splenomegaly. However, in certain individuals, as per in our case, EBV infection may lead to numerous acute complications and delayed effects. Morbilliform rashes can sometimes emerge following the administration of ampicillin to a patient with infectious mononucleosis, although the exact cause of this rash remains unclear. Moreover, splenic rupture, although rare, poses a potentially life-threatening risk and might present as the initial sign of IM that prompts the patient to seek medical attention. Furthermore, oral hairy leukoplakia can manifest as painless, white, and corrugated plaques typically affecting the sides of the tongue, particularly among individuals with advanced HIV infection. EBV infection is also linked to various lymphoproliferative disorders. Over a dozen specific gene mutations causing primary immune deficiency disorders have connections to severe EBV-induced diseases [66,67]. A study indicates that the onset and severity of infectious mononucleosis might be linked to prior infections with other viruses, like the influenza virus, which boosts the response of T cells to EBV infection [68]. Infectious mononucleosis stands out as the primary acute clinical presentation of EBV [64,65]. Typical symptoms include fever, pharyngitis, lymphadenopathy, and splenomegaly. However, in certain individuals, as per in our case, EBV infection may lead to numerous acute complications and delayed effects. Morbilliform rashes can sometimes emerge following the administration of ampicillin to a patient with infectious mononucleosis, although the exact cause of this rash remains unclear. Moreover, splenic rupture, although rare, poses a potentially life-threatening risk and might present as the initial sign of IM that prompts the patient to seek medical attention. Furthermore, oral hairy leukoplakia can manifest as painless, white, and corrugated plaques typically affecting the sides of the tongue, particularly among individuals with advanced HIV infection. EBV infection is also linked to various lymphoproliferative disorders. Over a dozen specific gene mutations causing primary immune deficiency disorders have connections to severe EBV-induced diseases [66,67]. A study indicated that the onset and severity of infectious mononucleosis might be linked to prior infections with other viruses, like the influenza virus, which boosts the response of T cells to EBV infection [68].

### 4.3. Laboratory Findings

EBV infection commonly displays hematologic abnormalities as a prominent laboratory feature. Typically, it showcases lymphocytosis, defined as an absolute count exceeding 4500/microL or, as observed on a peripheral smear, a differential count >50%. The smear might reveal significant atypical lymphocytes, representing >10% of the total lymphocytes. Predominantly, reactive lymphocytes in infectious mononucleosis patients belong to the CD8+ cytotoxic T cell category. It appears that the severity of the condition correlates with the extent of CD8+ lymphocytosis, as well as the EBV load in the blood [69]. Patients typically exhibit a total white blood cell count averaging between 12,000 to 18,000/μL, although some cases may present much higher counts. Certain patients might experience mild relative and absolute neutropenia and thrombocytopenia. These conditions are generally benign and tend to resolve on their own [70]. Less common hematologic manifestations encompass hemolytic anemia, thrombocytopenia, aplastic anemia, thrombotic thrombocytopenic purpura/hemolytic-uremic syndrome, and disseminated intravascular coagulation. Some of these complications arise due to EBV-triggered production of antibodies targeting red blood cells, white blood cells, and platelets. Primary EBV infection is a known initiator of hemophagocytic lymphohistiocytosis, an infrequent disorder characterized by cytopenias, abnormalities in liver function, coagulopathies, elevated serum ferritin levels, and other indications of substantial systemic inflammation. Liver function tests commonly reveal elevated aminotransferase levels in the majority of patients, but these tend to resolve on their own. Abnormalities in liver function tests in a patient with pharyngitis strongly indicate the possibility of EBV infection as a diagnosis.

### 4.4. Treatment and Prevention

Primary EBV infections typically require supportive therapy and little else. Treatment mainly involves managing symptoms for individuals with infectious mononucleosis. Fever, throat discomfort, and malaise can be addressed with acetaminophen or nonsteroidal anti-inflammatory drugs. Ensuring proper hydration and nutrition is crucial. The use of corticosteroids remains a debate. Previous data have shown that combining acyclovir and prednisolone reduced viral shedding in the throat and caused a reduction in lymphoid or mucosal swelling but did not shorten symptom duration or hasten return to school or work [71,72]. A subsequent analysis of seven studies did not find enough evidence to support steroid use for symptom relief, while two studies reported severe complications in patients given corticosteroids compared to those given a placebo [73]. As EBV generally resolves on its own, routine corticosteroid therapy is not recommended. However, corticosteroids might be considered for the management of certain EBV-related complications.

Studies have explored the use of intravenous and oral forms of acyclovir for treating acute EBV infections [71,74,75]. Although oropharyngeal shedding of the virus notably decreased during therapy with acyclovir, this effect was not sustained three weeks after treatment cessation. These findings align with the limited evidence showing the role of ongoing viral replication in the symptomatic EBV-induced mononucleosis phase. In many EBV-related malignancies where the virus life cycle stage has been identified, there is little evidence of permissive (lytic) infection. As acyclovir primarily inhibits linear EBV DNA replication, its use in diseases linked with latent infection might offer limited benefits. However, anecdotal evidence supports acyclovir use in EBV-induced hemophagocytic lymphohistiocytosis cases where replicating EBV was detected [76]. Some reports suggest the use of interleukin-2, interferon alfa, and intravenous immunoglobulins in EBV-related diseases. However, clear benefits of these approaches have not been widely established, except for potential efficacy in lymphomatoid granulomatosis and post-transplant lymphoproliferative disease (PTLD) [77]. A comprehensive analysis of 4466 allogeneic hematopoietic stem-cell transplant (HSCT) patients revealed that over two-thirds of patients with EBV-related PTLD survived following rituximab-based treatment, with survival rates influenced by factors such as age, extralymphoid tissue involvement, acute GVHD, and the management of immunosuppression [78]. Additionally, a retrospective study on the same patient population showed promising results, with an 84.5% overall response rate, 73.1% complete remission rate, and notably higher survival rates in patients with probable EBV disease compared to those with PTLD [79]. Evaluation of adoptive cell therapy involving EBV-specific cytotoxic T lymphocytes is ongoing for patients with EBV-associated lymphoproliferative disorders and malignancies [80]. In 2015, the US Food and Drug Administration recognized EBV-CTL as a breakthrough therapy for treating refractory EBV-post-transplant lymphoproliferative disorders. 

For individuals with active EBV infection, such as in primary or chronic active cases, adopting measures like frequent handwashing and avoiding the sharing of eating utensils, drinking glasses, and toothbrushes may help lower the risk of transmitting EBV to others. In patients undergoing solid organ or hematopoietic cell transplant, exposure to EBV can sometimes be prevented by choosing EBV-naïve donors for recipients who are also EBV-naïve. However, this is not always feasible. Hence, patients are usually monitored for EBV infection, and if EBV viremia is detected, strategies to minimize the risk of post-transplant lymphoproliferative disease are implemented. The strong evidence linking EBV to various human cancers has raised interest in developing a viral-based vaccine effective against these cancers. Glycoprotein (gp) 350/220, found abundantly in lytically infected cell plasma membranes and as the predominant protein on the virus coat’s outer surface, binds to the CD21 receptor on B cells, initiating infection. Additionally, a significant portion of the human EBV neutralizing antibody response targets gp350/220 [81]. Consequently, gp350/220 has been the primary EBV lytic-cycle gene being explored for a subunit vaccine. In animal trials, immunization with partially purified gp350/220 antigen led to EBV-neutralizing antibody production, protecting some cotton-top tamarins from a typically lethal EBV-induced lymphoma challenge [82]. Clinical trials investigating a recombinant EBV subunit gp350 vaccine demonstrated its safety and ability to induce an immune response [83]. Although it did not prevent EBV infection, the vaccine did reduce clinical symptoms.

### 4.5. Prognosis

The majority of individuals experiencing primary Epstein-Barr virus infection tend to recover without complications and develop long-lasting immunity. Typically, acute symptoms subside within one to two weeks; however, fatigue and reduced functional abilities may persist for months [84,85]. Approximately 10% of individuals continue to experience fatigue six months after the onset of symptoms [85,86]. However, this rate decreases in the following months, with most people eventually making a complete recovery. Some studies indicate that the severity of the initial illness may be linked to persistent fatigue [85,86], with female gender and pre-existing mood disorders being associated with a higher likelihood of experiencing prolonged fatigue [87]. The precise reasons why certain patients do not fully regain their previous health remain unclear, although abnormalities in mitochondrial function and the levels of messaging by regulatory molecules has been noted [88].

## 5. Conclusions

Acute acalculous cholecystitis is a benign complication of primary EBV infection, possibly linked to Gilbert syndrome, not requiring surgical intervention, and is commonly seen in young girls and women. Its pathogenesis is not fully ascertained. EBV infection should always be considered in the differential diagnosis of AAC.

This literature review provides a comprehensive overview of acalculous cholecystitis as a complication of EBV infection. The identified trends offer valuable guidance for clinicians in the diagnosis and management of this rare but significant condition. The present case adds to the growing body of literature and serves as a reminder of the heterogeneous nature of this disease, highlighting the need for a high index of clinical suspicion for timely diagnosis and intervention. Future research should aim to elucidate the underlying mechanisms contributing to this complication.

## Figures and Tables

**Table 1:** Laboratory tests follow-up.

Laboratory Test	Day of Admission Values	3rd Hospitalization Day	12th Day after Admission
Total bilirubin	2.8 mg/dL	5 mg/dL	1.6 mg/dL
Direct bilirubin	2.4 mg/dL	4.1 mg/dL	1 mg/dL
AST	468 U/L	457 U/L	214 U/L
ALT	423 U/L	414 U/L	196 U/L
ALP	667 U/L	696 U/L	350 U/L

Abbreviations: AST: aspartate aminotransferase; ALT: alanine aminotransferase; ALP: alkaline phosphatase. mg/dL indicates milligrams per deciliter, and U/L signifies units per liter.

## Supplementary Materials

The following supporting information can be downloaded at: https://www.mdpi.com/article/10.3390/v16030463/s1. Table S1: Methodological Quality Assessment Tool for Case-Reports/Series by Murad et al.; Table S2: Methodological Quality Assessment of Included Case Reports and Case Series Using the Tool, suggested by Murad et al. [8], Imad, H. A., et al. [89] and Guri A, et al. [90].
