# Supplementary material for: Acalculous Cholecystitis as a Complication of Primary Epstein-Barr Virus Infection: A Case-Based Scoping Review of the Literature
Source: Viruses. 2024 Mar 18;16(3):463. doi: 10.3390/v16030463 (PMC10974004; doi:10.3390/v16030463)
Supplement: Supplementary file 1 [file viruses-16-00463-s001.zip › viruses-2921983-supplementary.pdf]

Table S1. Methodological Quality Assessment Tool for Case-Reports/Series by Murad et al. [8]

Domain	Leading Explanatory Questions	Assessment Criteria
Selection	Does the patient(s) represent the whole experience of the investigator (center), or is the selection method unclear?	Yes/No
Ascertainment	Was the exposure adequately ascertained?	Yes/No
	Was the outcome adequately ascertained?	Yes/No
	Were other alternative causes that may explain the observation ruled out?	Yes/No
Follow-up	Was follow-up long enough for outcomes to occur?	Yes/No
Reporting	Is the case(s) described with sufficient details to allow other investigators to replicate the research or practitioners to make inferences related to their own practice?	Yes/No

Table S2. Methodological Quality Assessment of Included Case Reports and Case Series Using the Tool Suggested by Murad et al. [8]

Study Reference	Patient Selection	Ascertainment of EBV Infection	Ascertainment of AAC	Ruling Out Alternative Causes	Sufficient Follow-up	Detailed Reporting	Inclusion in the Review
Fretzayas A.et al [4]	Yes	Yes	Yes	Yes	Yes	Yes	Yes
Beltrame	Yes	Yes	Yes	Yes	Yes	Yes	Yes

V.et al [9]							
Attilakos A.et al [10]	Yes	Yes	Yes	Yes	Yes	Yes	Yes
Arya S.O.et al [11]	Yes	Yes	Yes	Yes	Yes	Yes	Yes
Suga K.et al [12]	Yes	Yes	Yes	Yes	Yes	Yes	Yes
Gagneux-BrunonA.et al [13]	Yes	Yes	Yes	Yes	Yes	Yes	Yes
Iaria C.et al [14]	Yes	Yes	Yes	Yes	Yes	Yes	Yes
Prassouli A.et al [15]	Yes	Yes	Yes	Yes	Yes	Yes	Yes
Cholongitas E.et al [16]	Yes	Yes	Yes	Yes	Yes	Yes	Yes
Lagona E.et al [17]	Yes	Yes	Yes	Yes	Yes	Yes	Yes
Ono S.et al [18]	Yes	Yes	Yes	Yes	Yes	Yes	Yes
Majdalani M.et al [19]	Yes	Yes	Yes	Yes	Yes	Yes	Yes
Koufakis T.et al [20]	Yes	Yes	Yes	Yes	Yes	Yes	Yes
Rodà D.et al [21]	Yes	Yes	Yes	Yes	Yes	Yes	Yes
Branco L.et al [22]	Yes	Yes	Yes	Yes	Yes	Yes	Yes
Pawłowska-Kamieniak A.et al [23]	Yes	Yes	Yes	Yes	Yes	Yes	Yes
Alkhoury F.et al [24]	Yes	Yes	Yes	Yes	Yes	Yes	Yes
Agergaard J.et al [25]	Yes	Yes	Yes	Yes	Yes	Yes	Yes
Koch A.	Yes	Yes	Yes	Yes	Yes	Yes	Yes

D.et al [26]							
Yang H. N.et al [27]	Yes	Yes	Yes	Yes	Yes	Yes	Yes
Rezkallah KN.et al [28]	Yes	Yes	Yes	Yes	Yes	Yes	Yes
Yoshie K.et al [29]	Yes	Yes	Yes	Yes	Yes	Yes	Yes
Pelliccia P.et al [30]	Yes	Yes	Yes	Yes	Yes	Yes	Yes
Hagel S.et al [31]	Yes	Yes	Yes	Yes	Yes	Yes	Yes
Nagdev A.et al [32]	Yes	Yes	Yes	Yes	Yes	Yes	Yes
Carrascosa MF.et al [33]	Yes	Yes	Yes	Yes	Yes	Yes	Yes
Strehle E.et al [34]	Yes	Yes	Yes	Yes	Yes	Yes	Yes
Sheybani F.et al [35]	Yes	Yes	Yes	Yes	Yes	Yes	Yes
Yesilbag Z.et al [36]	Yes	Yes	Yes	Yes	Yes	Yes	Yes
Cameron A.et al [37]	Yes	Yes	Yes	Yes	Yes	Yes	Yes
Höhn P.et al [38]	Yes	Yes	Yes	Yes	Yes	Yes	Yes
Boninsegna S.et al [39]	Yes	Yes	Yes	Yes	Yes	Yes	Yes
Young C.et al [40]	Yes	Yes	Yes	Yes	Yes	Yes	Yes
Ntelis K. et al [41]	Yes	Yes	Yes	Yes	Yes	Yes	Yes
Suda T.et al [42]	Yes	Yes	Yes	Yes	Yes	Yes	Yes
Langenohl	Yes	Yes	Yes	Yes	Yes	Yes	Yes

R. et al [43]							
Leganés Villanueva C. et al [44]	Yes	Yes	Yes	Yes	Yes	Yes	Yes
Nakagawa H. et al [45]	Yes	Yes	Yes	Yes	Yes	Yes	Yes
Harvey KG. et al [46]	Yes	Yes	Yes	Yes	Yes	Yes	Yes
Rein J.et al [47]	Yes	Yes	Yes	Yes	Yes	Yes	Yes
Avcu G.et al [48]	Yes	Yes	Yes	Yes	Yes	Yes	Yes
Celik F.et al [49]	Yes	Yes	Yes	Yes	Yes	Yes	Yes
Teopoulos Lamprianidis K. et al [50]	Yes	Yes	Yes	Yes	Yes	Yes	Yes
Barkho F. et al [51]	Yes	Yes	Yes	Yes	Yes	Yes	Yes
Trbojević T.et al [52]	Yes	Yes	Yes	Yes	Yes	Yes	Yes
Teles H.et al [53]	Yes	Yes	Yes	Yes	Yes	Yes	Yes
Khan U et al [54]	Yes	Yes	Yes	Yes	Yes	Yes	Yes
**Imad, H. A., et al [89]**	Yes	Yes	Yes	No	Yes	Yes	No
**Guri A, et al [90]**	Yes	Yes	No	Yes	Yes	Yes	No
